# Comparative Transcriptome, Metabolome, and Ionome Analysis of Two Contrasting Common Bean Genotypes in Saline Conditions

**DOI:** 10.3389/fpls.2020.599501

**Published:** 2020-12-10

**Authors:** Harun Niron, Nazire Barlas, Bekir Salih, Müge Türet

**Affiliations:** ^1^Department of Molecular Biology and Genetics, Bogazici University, Istanbul, Turkey; ^2^Department of Chemistry, Hacettepe University, Ankara, Turkey

**Keywords:** common bean, metabolome, *Phaseolus vulgaris* L., salt-stress, tolerance, transcriptome

## Abstract

Soil salinity is a major abiotic stress factor that limits agricultural productivity worldwide, and this problem is expected to grow in the future. Common bean is an important protein source in developing countries however highly susceptible to salt stress. To understand the underlying mechanism of salt stress responses, transcriptomics, metabolomics, and ion content analysis were performed on both salt-tolerant and susceptible common bean genotypes in saline conditions. Transcriptomics has demonstrated increased photosynthesis in saline conditions for tolerant genotype while the susceptible genotype acted in contrast. Transcriptome also displayed active carbon and amino-acid metabolism for the tolerant genotype. Analysis of metabolites with GC-MS demonstrated the boosted carbohydrate metabolism in the tolerant genotype with increased sugar content as well as better amino-acid metabolism. Accumulation of lysine, valine, and isoleucine in the roots of the susceptible genotype suggested a halted stress response. According to ion content comparison, the tolerant genotype managed to block accumulation of Na^+^ in the leaves while accumulating significantly less Na^+^ in the roots compared to susceptible genotype. K^+^ levels increased in the leaves of both genotype and the roots of the susceptible one but dropped in the roots of the tolerant genotype. Additionally, Zn^+2^ and Mn^+2^ levels were dropped in the tolerant roots, while Mo^+2^ levels were significantly higher in all tissues in both control and saline conditions for tolerant genotype. The results of the presented study have demonstrated the differences in contrasting genotypes and thus provide valuable information on the pivotal molecular mechanisms underlying salt tolerance.

## Introduction

Salt accumulation has become one of the most imminent agricultural threats in the recent years. It is estimated that nearly 20% of cultivated and 33% of irrigated farmland has been affected by salt globally (Munns and Tester, 2008). These percentages are expected to increase annually through diverse causes such as excessive evaporation, improper irrigation, or inadequate precipitation, which are related to the global climate change. At the current rate of salt accumulation, 50% of arable farmlands may become salt-affected by 2050 (Jamil et al., 2011). Most of the economically significant crops such as rice, maize, potato, tomato, and legumes are rather susceptible to salinity (Muchate et al., 2016). Production of these and other crops will have to increase up to 70% to cope up with the steadily growing human population, which is predicted to surpass 9 billion by 2050 (Davies and Bowman, 2016). These global challenges call for urgent but sustainable solutions, which can be found in more-salt tolerant varieties of cultivated plants. The genetic program for salt tolerance can be transferred to salt-sensitive crops with otherwise valuable agronomical traits using conventional breeding or transgenic methods (Hanin et al., 2016). Therefore, identification of genes responsible for the superior salt tolerance, their functional characterization, and understanding of the associated metabolic processes are essential for sustainable agriculture in the era of overpopulation and climate change.

A soil is considered saline if it contains enough soluble salt to have detrimental effects on the growth of crop plants. Saline soil is roughly defined as a soil with electrical conductivity of saturated extract (ECe) equal or higher than 4 dS m-1 (Shrivastava and Kumar, 2015); but the majority of common crop yields display reduction even at lower ECes (Munns, 2005; Jamil et al., 2011). Although various salts contribute to soil salinity, sodium chloride (NaCl) is the most predominant form. Elevated NaCl disrupts diverse systems necessary for consistent plant growth and development (Munns and Tester, 2008; Shahzad et al., 2013). It leads to two types of generalized stress for plants: osmotic and ionic stress. Osmotic stress arouses from the decreased water potential and water availability for the plant. Ionic stress, on the other hand, is caused by the toxic ion accumulation over time (R. Munns, 2002). These stress factors create a network of restraints on survival, not just with ion toxicity and water retention, but also with nutrient and metabolic imbalance that collectively become a physiological response (Tester and Davenport, 2003).

Understanding this response is a laborious task and requires a comprehensive strategy against these stress factors. Essentially, plants employ water homeostasis control and adjustment of osmotic balance, salt exclusion, sequestration, oxidative protection, regulation of potassium exchange, biochemical responses, and growth regulation to cope with salt stress (Tester and Davenport, 2003; Munns, 2005; Munns and Tester, 2008; Shabala and Cuin, 2008). As there is a complex network of connections between these systems, tolerance to salt may involve the regulation of thousands of genes (Monforte et al., 1997; Foolad, 2004). This complexity can be reduced by making a comparison among species and varieties of plants that have evolved distinct mechanisms to deal more successfully with salinity. Although tolerant and susceptible plants use very similar strategies, differential regulation of the responses can indicate the key points of the salt tolerance mechanisms (Nilsen et al., 1996).

Common bean (*Phaseolus vulgaris* L.) is a grain legume with substantial agricultural importance. As a rich source of vitamins, minerals, and dietary proteins it is an essential nutrient for human consumption. It represents approximately half of the produced and consumed grain legumes in the world (Broughton et al., 2003). With its high phytochemical content and high antioxidant capacity, it supports the human immune system against disorders like obesity, cancer, and cardiovascular diseases (Pinheiro et al., 2010). Moreover, it is integral to sustainable agriculture as it supplements the soil with nitrogen through symbiotic associations (Broughton et al., 2003). However, common bean is fairly susceptible to salt. This crop can suffer nearly 20% yield loss even in slightly saline soil with 1 dS m-1 ECe (Chinnusamy et al., 2005). This inconvenience can be mitigated through somewhat salt tolerant genotypes of common bean cultivars such as Ispir, but this solution may not be sustainable against the increasing soil salt content. To expand the tolerance capacity in such superior varieties, it is necessary to understand the existing mechanisms, so that potential paths to further improvement become unveiled.

A phenotype is the product of dynamic interplay between various factors such as DNA, RNA, proteins, and metabolites together with the influence of the environment. Therefore genome- and transcriptome-based approaches demand studies from complementary fields such as proteomics and metabolomics to establish accurate genotype–phenotype relationships (Arbona et al., 2013). While gene and protein expression exhibit capacity and inclination of a plant in response to environmental conditions, metabolite content forms the link between expression and environment (Arbona et al., 2013).

In this study, we have compared two genotypes of *P. vulgaris* L. that contrast in their response to salt stress, namely Ispir (tolerant) and TR43477 (susceptible) (Dasgan and Koc, 2009). This comparative analysis facilitates an in-depth understanding of salt stress tolerance mechanisms by combining transcriptomics, metabolomics and ionomics data under salinity conditions. Our results indicated differentially regulated transcripts that can be further functionally characterized by mutagenesis-based approaches. Our data also revealed the enriched and depleted metabolic pathways and their disparity in these genotypes. We believe, this study will also provide insights into the genetic programs and the regulation of other abiotic stress responses, since they are known to share mechanisms (Zhu, 2016).

## Materials and Methods

### Plant Growth, Salt-Stress Application, and Sample Collection

Ispir (salt-tolerant) and TR43477 (salt-susceptible) varieties of common bean (*Phaseolus vulgaris* L.) were grown and salt-treated in hydroponic conditions to collect tissue samples. The seeds were sterilized in 5% hypochlorite solution. Germination was performed in vermiculite containing plug trays under a 16-h light/8-h dark photoperiod at 24°C/20°C cycle with 50–70% relative humidity. Trays were irrigated daily with 1X Hoagland nutrient solution (Hoagland and Arnon, 1950) until the plants got fully expanded foliage (Nine days for Ispir and eight days for TR43477 after sowing). Seedlings from each genotype were transferred to hydroponics system. Salt treatments were carried out in the same conditions as in our earlier transcriptome study Hiz et al. (2014). Gradual step acclimation method was employed to prevent osmotic shock (Sanchez et al., 2008). Five days post transfer, the plants were subjected to gradual NaCl treatment starting with 50 mM first day, increased to 100 mM on the second day, and set to 125 mM on the third day. In total, the plants were grown under 125 mM NaCl for three days before they were sacrificed for tissue sample collection.

### RNA-Sequencing and Transcriptome Analysis

Total RNA extractions from the leaf and root tissues of three plants as biological replicates for control and salt-treatment conditions separately, were performed with RNeasy Plant RNA extraction kit (QIAGEN, United States). Sample qualities were inspected with Agilent 2100 Bioanalyzer system by measuring the RNA integrity number (Schroeder et al., 2006). Poly(A +) enrichment and cDNA library construction were performed with Truseq Stranded mRNA kit (Illumina, United States). The obtained paired-end library was sequenced using NovaSeq 6000 system. Raw data quality control was performed with FastQC tool (Andrews and Babraham Bioinformatics, 2010) and Trimmomatic software (Bolger et al., 2014) was employed for raw-read trimming. Genome indexing and paired read alignment were performed with HISAT2 tool (Kim et al., 2015) using *Phaseolus vulgaris* genome v.2.1 (phytozome.org) as reference. Read counts, determined with Seqmonk v.1.44.0 tool^1^, were harnessed with EdgeR (McCarthy et al., 2012) for differential expression analysis. Differentially expressed genes (DEGs) were selected among all genes with a filter of |log_2_ fold change| > 1 and FDR < 0.01. DEGs from different genotypes were further subjected to intensity difference filter of Seqmonk v.1.44.0 tool, which compares the datasets and detects the differences with lowest variability, to discover the genes that displayed highly reliable and sharp changes in response to salt treatment.

#### Verification of Expressional Levels With qRT-PCR Analysis

Ten genes were selected for the qRT-PCR analysis: The procedure was carried out with 10 ng of cDNA from the roots of each variety per reaction. Three technical replicates were performed for each of the three-biological replicates. PikoReal 96 Real-time PCR system (Thermo Fisher Scientific, DE) was utilized for the experiment. *Actin-11* (GenBank: CV529679.1) and *insulin-degrading enzyme* (GenBank: FE702602.1) genes of common bean were used as the reference genes as they were reported to have stable expression under salt treatment in common bean (Borges et al., 2012). Relative expression levels were calculated by 2^ΔΔ^ Ct method (Livak and Schmittgen, 2001). The correlation between RNA-Seq and qRT-PCR results was assessed with Pearson correlation coefficient. Primers that were used in this study can be found in Table 1.

**TABLE 1:** qRT-PCR primers used for RNA-seq validation.

Sequence	Transcript ID	Amplicon size	Annealing T. (°C)
TCTTGCCTTGATCTTCGG	Phvul.001G195700	172	53
AGGTTTGAATAGAGGATGTG			
ACTCCAACAAACTCGAAACA	Phvul.002G027900	234	55
CACATACCACTCGGACCA			
TGATCCCATTGCAAATCC	Phvul.008G170800	151	53
TCCCCCCATAAAACCAAC			
CTCCACCTTTTCCACCAAC	Phvul.009G105300	157	56
CTTCCCACTACTCCTATTCC			
GCTATGGTTCCAGCTTTT	Phvul.006G159600	120	54
AGTTATTGGGGTTGGGTT			
CTCCTTTATCGCCTTCCT	Phvul.001G083000	230	54
ACTTCCGCATTACCAACA			
GCCTTCTCTTTTACCTTCT	Phvul.004G117100	102	53
ACACCACCATAATCCTCA			
GCTAGCTGTTCCATTTACGCAGAGT	Phvul.003G229500	100	60
AGCTGCCGTAGAGTTTGATTGCACC			
GCAGCTCCCAACCACTGACTAC	Phvul.001G181100	186	58
CCATCCAACCAAAGATCAACGCCCA			
AACCATGCCTTCACCAGCTTCAAAT	Phvul.005G051600	107	60
AGGTTGTGGGAGAAGAAGATGTGGA			
TGCATACGTTGGTGATGAGG	Phvul.008G011000 (Actin-11)	190	58
AGCCTTGGGGTTAAGAGGAG			

#### Gene Ontology (GO) and KEGG Pathway Enrichment Analyses

Gene ontology IDs for the transcripts were obtained from the Biomart (Smedley et al., 2015). GO enrichment analysis was performed with the GO-IDs of DEGs via AgriGO v2.0 tool (Tian et al., 2017). KEGG pathway enrichment analysis was performed with the transcript IDs via ShinyGo v.0.61 (Ge et al., 2020). In both enrichment analyses, the enriched terms were subjected to multi-test adjustment with Benjamini-Hochberg method (Benjamini and Hochberg, 1995) and terms with FDR < 0.05 were selected.

### Untargeted Metabolomics

#### Extraction of Metabolites

The metabolite extraction procedure for the leaf and root tissues of five biological replicates for control and salt-treatment conditions separately, was performed as described by Lisec et al. (2006). 100 mg of flash-frozen ground tissue samples were mixed with 60 μl of water containing ribitol as internal standard. The samples were mixed with 0.3 ml of methanol and 0.1 ml of chloroform and vortexed for 5 min followed by incubation at 70°C for 10 min. After centrifugation, supernatants were collected to be dried in a vacuum-dryer system. Following desiccation, each sample was incubated for 2 h at 37°C with 80 μl of methoxamine hydrochloride. Derivatization for gas-chromatography was performed with 1% trimethylchlorosilane (TMCS) in N-Methyl-N-(trimethylsilyl)-trifluoroacetamide (MSTFA) (100 μl) at 70°C for 1 h (Lisec et al., 2006).

### Gas Chromatography-Coupled Mass-Spectrometry

Untargeted metabolomics analysis was carried out with Gas Chromatography system (Agilent technologies 6890 N Network GC system, United States) and Mass Spectrometry system (Agilent technologies 5973 inert mass selective detector, United States) equipped with automatic injector (Agilent Technologies 7683 series, United States). For the ionization of the compounds, Electron Impact (EI) ionization source was used in positive ion mode at 70 electron-volts. Al the parameters for GC-MS system used in this study are given in Table 2.

**TABLE 2:** Conditions for GC-MS measurements.

Gas Chromatography		Mass Spectrometry	
Parameter	Type and Set Value	Parameter	Set Value
Column Name	HP-5MS	Solvent delay	2.5 min.
Split ratio	01:25	Low mass (amu)	70
Carrier gas	He	High mass (amu)	600
Actual length (m)	30	Aux. temperature (°C)	250
Internal diameter (mm)	250	Acquisition mode	Scan
Film thickness (mm)	0.25	Electron multiplier voltage (V)	2282
Inlet temperature (°C)	230	Ion Source temperature (°C)	180
Inject volume (ml)	1	Quadrupole temperature (°C)	230
Temperature programming	Start with 80°C and set 2 min, up to 300°C with 15°C/min ramp and then set 10 min. at 300°C. Total run time for each sample is ∼ 27 min.	Mass tuning compound	Perfluorotributylamine (PFTBA)

#### GC-MS Data Analysis

In-house MassHunter WorkStation with MSD ChemStation DA software (Agilent, United States) was utilized for GC-MS data processing such as transformation of the retention time, chromatogram alignment, peak extraction, normalization, and annotation. Wiley7n, Nist98, and W9N11 libraries were utilized for compound identification. Similarity ratio of 90% was taken into consideration for software-suggested annotations and final compound annotations were selected under manual curation. Data normalization was performed with the default options of the software. Principal component analysis for dimensionality reduction of the normalized data was implemented with XLSTAT software (Addinsoft Corporation, United States) (Addinsoft, 2019). Differentially accumulated/depleted metabolites (DADMs) were determined by statistical significance (*p* < 0.05) according to univariate analysis (two-sample *t*-test).

### Extraction of Heavy Metals and Ionomics

Flash-frozen ground leaf and root tissue samples of five biological replicates for control and saline conditions were dried in oven at 80°C and 100 mg was mixed with 10 ml HNO_3_ and 5 ml H_2_O_2_ in 50 ml Falcon tubes. The digestion was implemented by heating the samples 10 min at 100°C, then 15 min at 150°C, and finally 15 min at 180°C. The solutions were completed to 25 ml with dH_2_O. Concentrations of six ions (B, Mn, Fe, Cu, Zn, Mo) were measured by inductively coupled plasma mass spectrometry (Agilent 7700 Series ICP-MS, Agilent, United States) and concentrations of other four ions (Na, K, Mg, Ca) were measured by inductively coupled atomic emission spectrometry (Agilent 700 Series ICP-OES, Agilent, United States). Differentially accumulated/depleted ions were determined by statistical significance (*p* < 0.05) according to two-sample t-test. Statistical significance of distinction between the responses of the genotypes was measured by two-way ANOVA with replication (*p* < 0.05) with Excel Analysis ToolPak add-inn (Microsoft, 2019).

### Omics Data Merge and Pathway Analysis

Transcriptomics, metabolomics, and ionomics data for differentially expressed genes, regulated metabolites, and ions in Ispir and TR43477 varieties were integrated via KEGG Mapper (Kanehisa and Goto, 2000). KEGG pathways were used for a pathway-based integration to generate a representative map of carbon and amino acid biosynthesis metabolisms.

#### Chlorophyll Content Measurement

Chlorophyll contents of the leaves of five biological replicates were determined as described by Warren, 2008. Specific absorbance values of methanol extracted pigments were used in equations (Ritchie, 2006) to estimate chlorophyll a, chlorophyll b, and carotenoid contents of the leaves.

## Results

### Transcriptome Analysis Signifies the Whole-Plant and Tissue-Specific Differences Upon Salt-Stress Treatment

#### Overview of RNA-Sequencing Results

**FIGURE 1:**
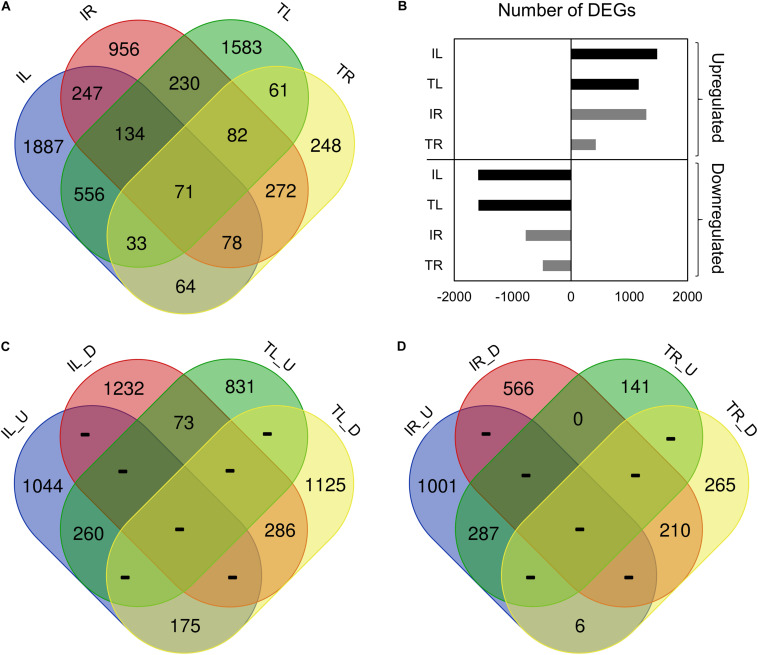
Venn diagram representation of the tissue- and genotype-specific DEGs distribution. IL_U: Ispir Leaf Upregulated; IL_D: Ispir Leaf Downregulated; TL_U: TR43477 Leaf Upregulated; TL_D: TR43477 Leaf Downregulated; IR_U: Ispir Root Upregularted; IR_D: Ispir Root Downregulated; TR_U: TR43477 Root Upregulated; TR_D: TR43477 Root Downregulated.

After adapter removal, reads had presented an average Q30 of 95.14%, with the lowest being 94.34%. On average, the mapping has produced the 89.05% concordant alignment and the 96.28% overall alignment to the reference (Table 3). The greatest number of DEGs was observed in Ispir leaves (IL) with 3072 genes, while roots of the TR43477 (TR) displayed the lowest number of DEGs, with 910 genes (Figures 1A,B). On the other hand, roots of Ispir (IR) and leaves of TR43477 (TL) displayed similar numbers of 2700 and 2750 DEGs, respectively.

**TABLE 3:** Statistics for raw read alignment to refence genome with HISAT2 tool.

			Paired read count	Concordant alignment (%)	Overall alignment (%)
ISPIR	LEAF	Control_1	15492583	92.1	97.99
		Control_2	24501307	89.49	97.34
		Control_3	24519671	92.88	97.92
		Treatment_1	14073496	91.15	97.25
		Treatment_2	22721821	90.4	97.52
		Treatment_3	14335336	92.2	97.85
	ROOT	Control_1	15713485	87.87	96.25
		Control_2	15019777	89.31	95.87
		Control_3	16496524	89.15	96.94
		Treatment_1	14253723	85.57	96.5
		Treatment_2	16537801	90.26	96.95
		Treatment_3	15648266	90.55	97.33
TR43477	LEAF	Control_1	15753609	88.56	95.54
		Control_2	15196921	90.44	96.38
		Control_3	17281002	88.51	96.04
		Treatment_1	14931185	88.20	95.71
		Treatment_2	18796243	88.77	95.67
		Treatment_3	17793700	88.73	95.66
	ROOT	Control_1	15559453	87.59	95.64
		Control_2	20006512	87.48	95.5
		Control_3	17229757	86.48	93.65
		Treatment_1	17897796	87.2	95.25
		Treatment_2	31045673	86.69	94.3
		Treatment_3	26924512	87.62	95.72

Comparison of the DEG lists has shown that 71 genes were differentially expressed in all tissues and both varieties upon salt treatment (Figure 1A). 3090 DEGs were specific to Ispir with 247 DEGs expressed in both above-ground and under-ground tissues. On the other hand, TR43477 displayed 1892 specific DEGs, only 61 of which were shared between tissues (Figure 1A). Regarding the number of upregulated and downregulated DEGs, IL displayed the highest number in both categories among all samples, while the lowest numbers in both categories were associated with TR (Figure 1B). Examination of upregulated and downregulated DEGs according to the tissue type demonstrated that leaves of the two genotypes shared 260 upregulated and 286 downregulated DEGs, whereas 248 genes displayed contrasting patterns (Figure 1C). The root tissues exhibited similar numbers of shared upregulated and downregulated DEGs, with 287 and 210 DEGs, respectively, but only six DEGs displayed contrasting pattern in roots (Figure 1D).

To check the reliability of RNA-Seq data, expression analysis of 10 genes was performed with qRT-PCR for the roots of both varieties. The results indicated high correlation levels with Pearson r values of 0.87 and 0.84 for the resistant (Supplementary Figure 1A) and the susceptible genotype, respectively (Supplementary Figure 1B).

#### GO and KEGG Pathway Enrichment Analysis of DEGs in Response to Salt-Stress

**FIGURE 2:**
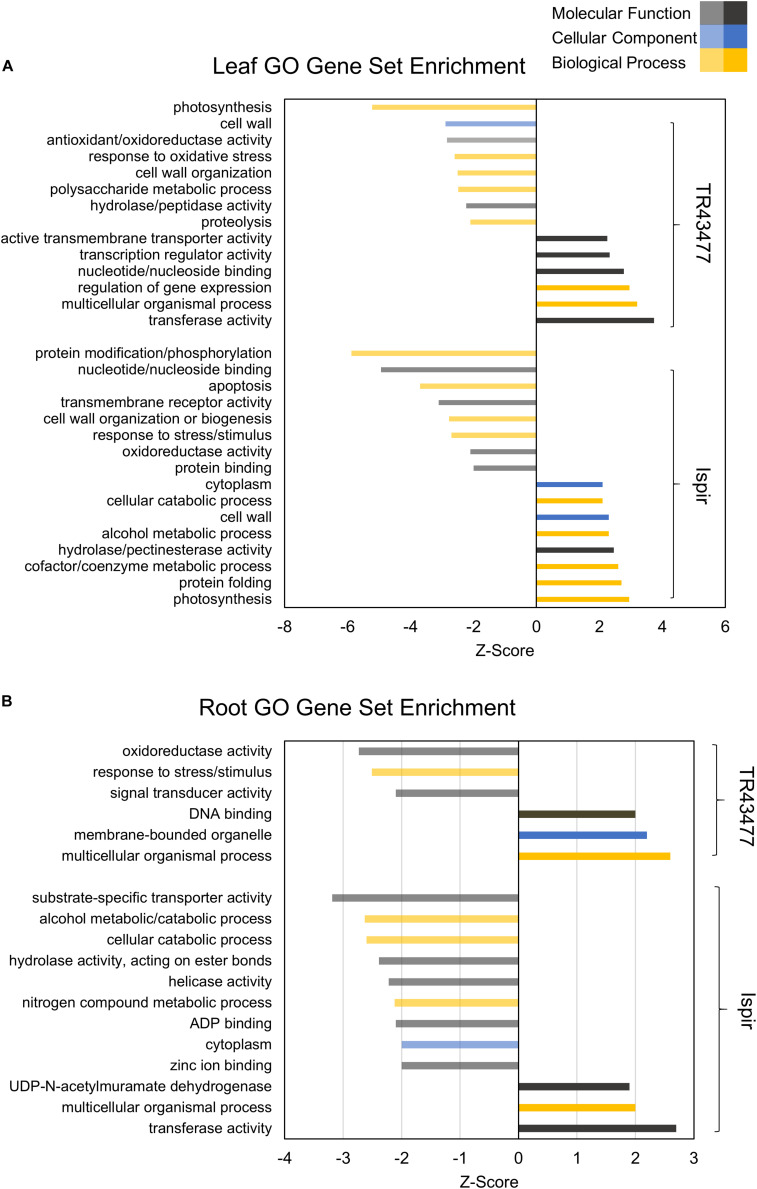
Parametric GO gene set enrichment of leaf **(A)** and root **(B)** DEGs. The Z-Score was derived from the number of DEGs and their log_2_FC. Performed for at least five genes in a gene set (adjusted *p*-value < 0.05).

**FIGURE 3:**
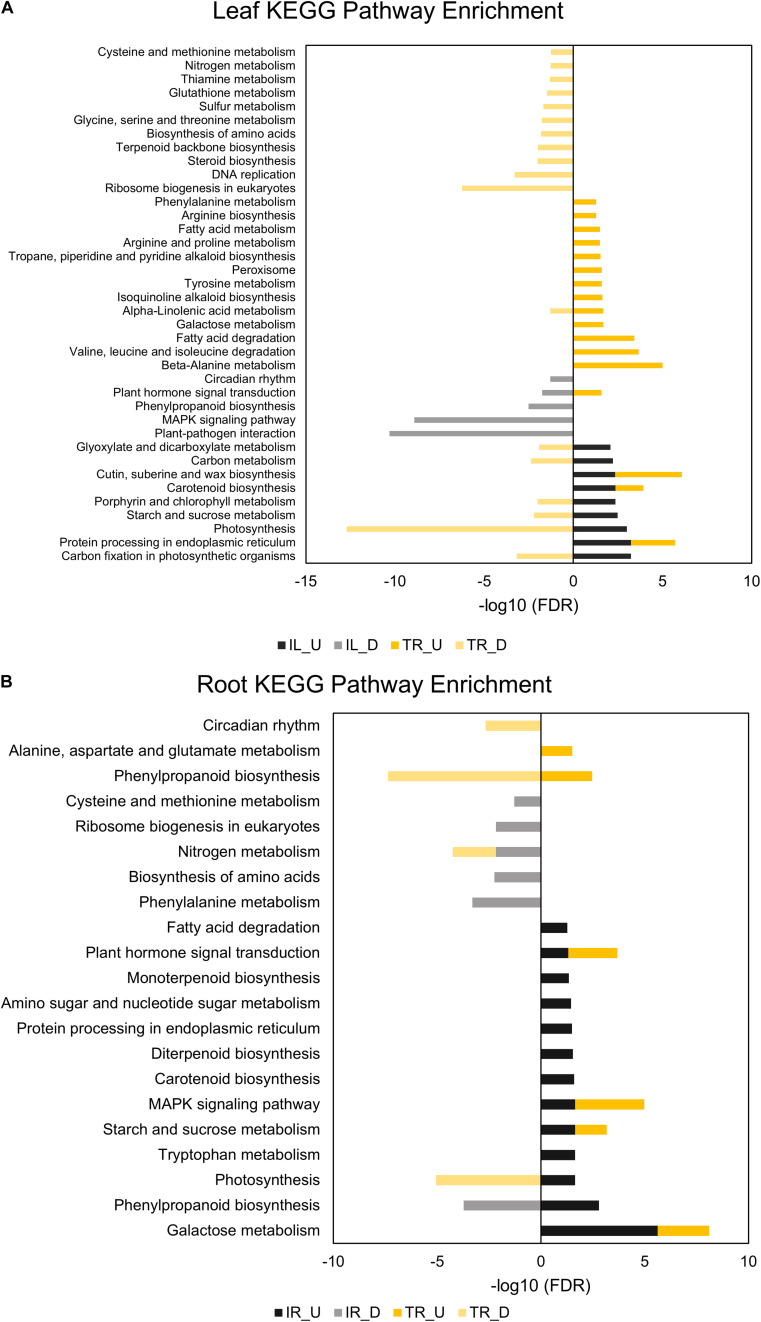
KEGG pathway enrichment analyses of leaf **(A)** and root **(B)** DEGs. “U” depicts upregulated and “D” depicts downregulated DEGs in the legends of KEGG graphs. Performed for at least five genes in a pathway (adjusted *p*-value < 0.05).

**FIGURE 4:**
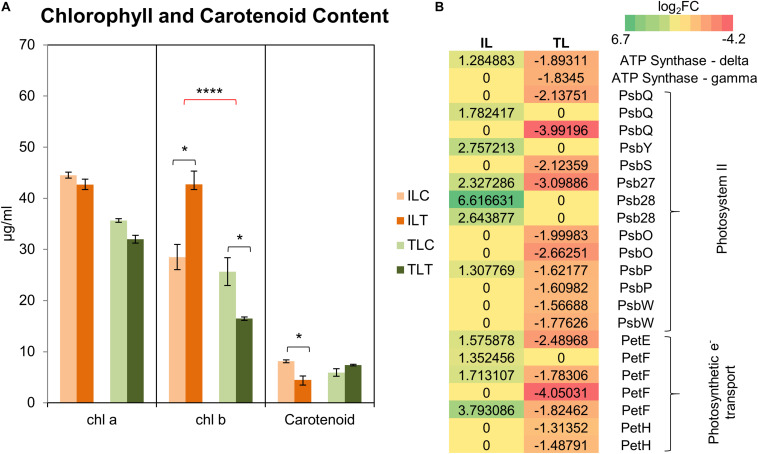
Chlorophyll and carotenoid content changes in the leaf tissues **(A)**. “C” depicts control condition and “T” depicts treatment condition. Heatmap with log_2_-fold changes of photosynthesis-related transcripts in KEGG pathways **(B)**. A more comprehensive table can be found at **Additional File 3**. * indicates significance and quantity of * displays the level of significance. (*p < 0.05; **p < 0.01; ***p < 0.005; ****p < 0.001).

Comparative GO and KEGG pathway enrichment analyses of DEGs (Figures 2, 3, **Additional Files 1, 2**) demonstrated distinct responses of these two genotypes against salinity stress. Photosynthesis-related terms were enriched in IL but depleted in TL according to both databases. KEGG results also indicated a similar trend for porphyrin and chlorophyll metabolism. This result was in concert with the leaf chlorophyll contents for IL and TL (Figure 4A): While IL *chl* b content has displayed a significant increase, TL *chl* b content decreased and the difference between the change was highly significant. Genes of photosystem II and photosynthetic e^–^ transport modules have demonstrated a strong contrast in salinity responsive regulation for those genotypes (Figure 4B; **Additional File 3**). Furthermore, *ATP synthase delta* subunit (Phvul.003G211100) was upregulated in IL but it was downregulated together with *ATP synthase gamma* subunit (Phvul.006G149700) in TL which implicated a disrupted proton conduction for the susceptible genotype together with decreased chlorophyll content (Figure 4A; **Additional File 3**).

TL was depleted in GO terms for polysaccharide metabolic process and KEGG terms for carbon fixation, whereas carbon fixation related GO terms and KEGG pathways were enriched in IL (Figures 2A, 3B). Sucrose and starch metabolism and glyoxylate and dicarboxylate metabolism displayed a similar result in KEGG pathway analysis: enriched in IL, depleted in TL (Figure 3A). Especially, Phvul.004G029100 (*Starch synthase*), Phvul.008G210100 (β-*D-glucan exohydrolase*) and Phvul.011G107700 (β-*amylase 5*) genes have stood out in IL for sucrose and starch metabolism in intensity difference analysis (**Additional File 4**). IL was depleted in GO terms related to transcription, translation, and post-translational modification, while transcriptional, expressional regulation, and functional modification-related GO terms were enriched in TL. In relation, TL displayed enrichment and depletion patterns for various amino acids together with a decrease in DNA synthesis and ribosome biogenesis-related terms. Cell wall and cytoplasm (cellular components)-related GO terms were enriched in IL, while GO terms related to manufacture of cell wall components were depleted in TL. IL displayed a complex response; diminished cell wall organization together with increased pectinesterase activity for cell-wall modification in GO analysis, while TL had diminished cell-wall organization (Figure 2A). Notably, IL displayed eight separate upregulated pectinesterase-related genes (**Additional File 3**). KEGG analysis indicated that both IL and TL were enriched in cutin, suberine, and wax biosynthesis. IL was enriched in protein folding activity and cofactor/coenzyme metabolism-related GO terms. TL, on the other hand, displayed diminished proteolysis and peptidase activities. Both IL and TL were enriched in KEGG terms for protein processing in endoplasmic reticulum (Figures 2A, 3A).

The responses of roots were limited compared to leaves: While TR was enriched in GO terms for transcriptional regulation, IR had enriched functional modification terms in GO and protein processing in endoplasmic reticulum pathway terms in KEGG. Indeed, five different heat shock family genes (Phvul.003G154800, Phvul.004G107700, Phvul.004G129400, Phvul.008G112700, and Phvul.009G080200) that are part of KEGG ‘Protein processing in endoplasmic reticulum’ pathway displayed intense upregulation patterns in IR tissue (**Additional File 4**) which hints the significant activity of unfolded or misfolded protein response. Moreover, IR had decreased helicase activity-related GO terms, probably an indication of halted DNA replication and modification, together with diminished GO nitrogen compound metabolic process terms, which is a sign of decreased translational activity. Although KEGG pathway results demonstrated that nitrogen metabolism was depleted in both IR and TL, IR was also depleted in biosynthesis of amino acids and ribosome biogenesis (Figures 2B, 3B). Concerning that, intensity difference filtering pointed out the downregulation of a *nicotinate phosphoribosyltransferase* homolog (Phvul.002G017800) that is part of GO ‘nitrogen compound metabolic process’ and ‘NAD metabolic process’ terms, together with the downregulation of a putative helix-loop-helix transcription factor (Phvul.001G126400) in IR (**Additional File 4**) which may be key constituents of difference in response between these genotypes.

### Metabolic Characteristics of the Tolerant and Susceptible Genotype Under Salt Stress

**FIGURE 5:**
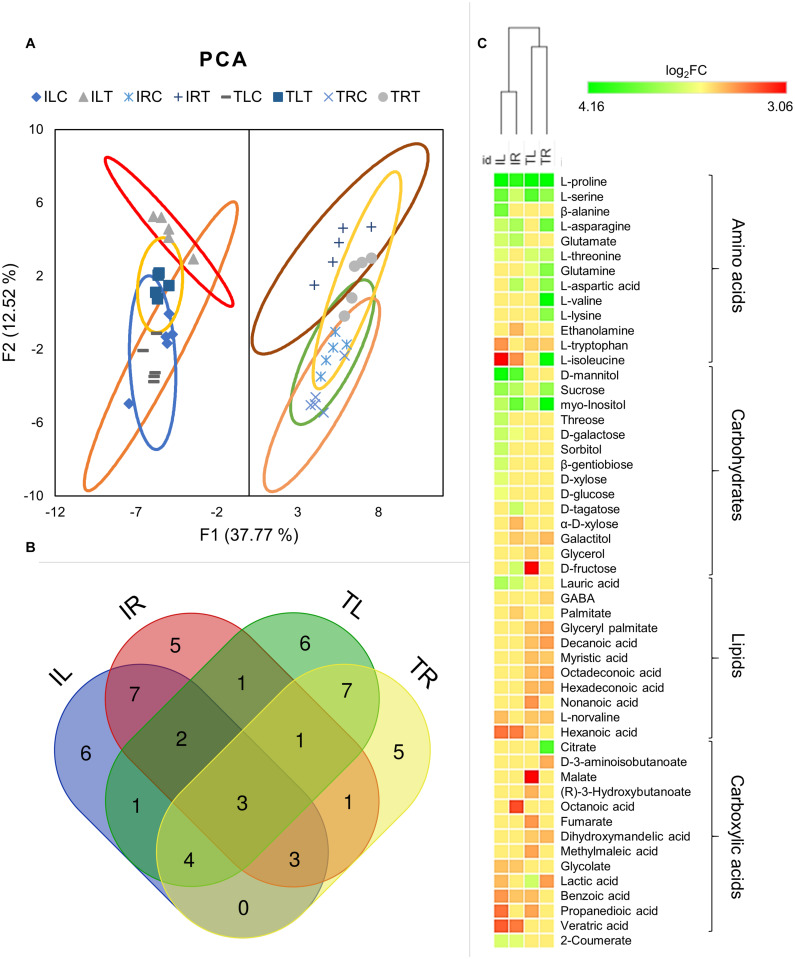
Principal component analysis was employed to visualize the distinction of metabolic responses in different tissues and conditions **(A)**. Venn diagram displays the distribution of DADMs among tissues in salt stress **(B)**. Hierarchical clustering analysis of DADMs demonstrated the differential reaction of the two genotypes **(C)**.

Untargeted GC-MS analysis has detected 79 different metabolites; 32 of which were Ispir-specific, and only 13 of which were TR43477-specific, while 34 metabolites were detected in both genotypes (**Additional Files 5, 6**). Principle component analysis (F1 and F2 represented a total of 50.3% of all data) of genotypes and tissues has clearly distinguished the behavior of leaf and root tissues from each other (Figure 5A; **Additional File 7**). The close projection of biological replicates indicated a reliable correlation for replicas. For both leaf and root tissues, Ispir has displayed a greater difference between control and stress-treated components compared to TR43477 (Figure 5A).

Although all the samples had similar numbers of differentially accumulated/decreased metabolites (DADMs), IL had the highest number of accumulated metabolites (17 metabolites) and TL had the highest number of decreased metabolites (19 metabolites). The number of accumulated and decreased metabolites were roughly even for both root tissues. Out of total 52 DADMs (number of metabolites significantly accumulated/decreased in at least one of the samples), only three (L-proline, L-serine, and myo-Inositol; all were accumulated) were found in all tissues and genotypes and 22 were unique to specific tissues and genotypes (Figure 5B).

Hierarchical clustering of DADMs displayed the distinction between the responses of different genotypes to salt treatment. The algorithm placed the root and leaf tissues of genotypes in the same clade and diverged the tissues of contrasting genotypes (Figure 5C). This analysis also demonstrated the difference of metabolic response patterns of tissues and genotypes; while IL has accumulated nine separate carbohydrates in response to salt, TL managed to accumulate only one and displayed decreased amounts for other three. A similar imbalance was also evident in the root tissues, as IR had six accumulated and two decreased carbohydrates, while TR displayed two accumulated and one diminished metabolite identified as a carbohydrate. (Figure 5C; Supplementary Figure 2). All tissues mainly increased their amino acid contents, but this increase was particularly noteworthy for TR as it displayed nine DADMs for the amino acid class. The lipid contents (mainly structural derivatives of a fatty acid, decanoic acid) of both root and leaf tissues of TR43477 were significantly decreased compared to tissues of Ispir. Carboxylic acid contents appeared to decrease in both tissues and both genotypes in response to salt stress. Also, accumulation of 2-coumerate was detected in both leaves and roots of Ispir (Figure 5C; Supplementary Figure 2).

### Transcriptional and Metabolic Changes in the Carbon and Amino Acid Metabolism

**FIGURE 6:**
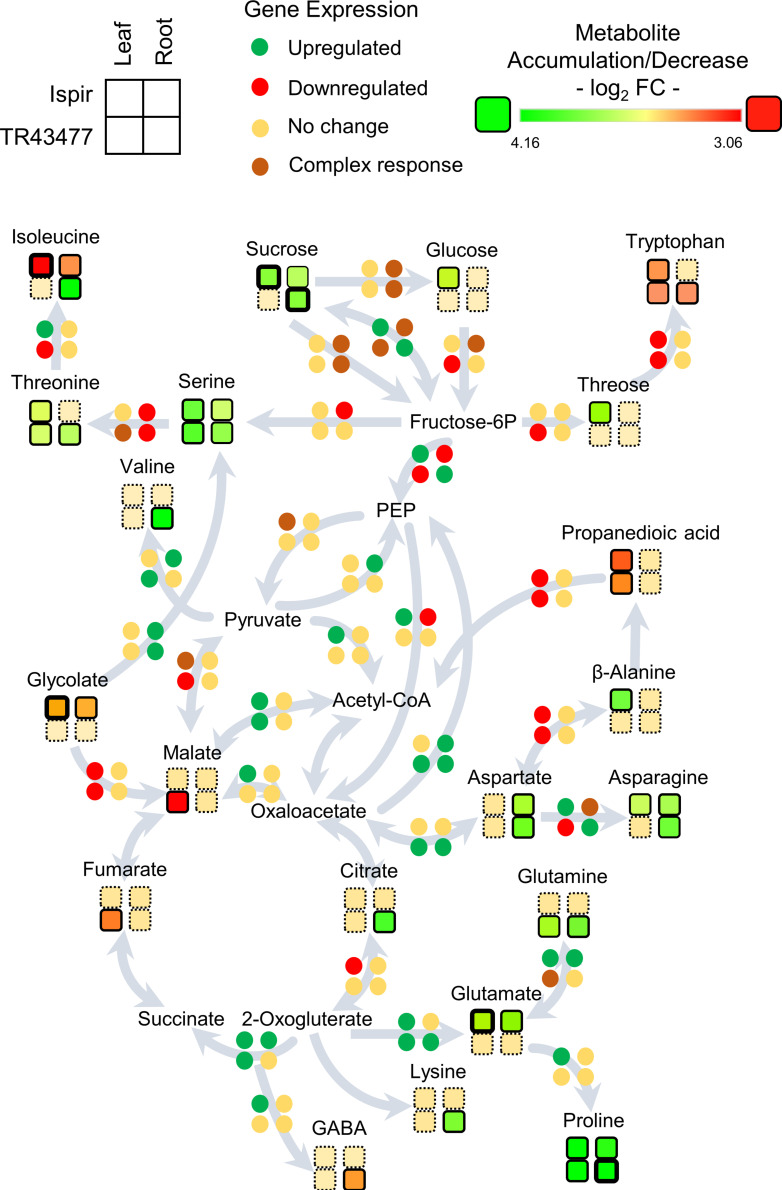
Representation of the transcript and metabolite changes in carbon and amino-acid metabolism in root and leaf tissues of both genotypes in saline conditions. The pathway map was derived from KEGG database. Disk units for gene expression represent one or more genes depending on the pathway: Detailed list of the relevant genes can be found in **Additional file 3**; Primary Metabolism tab. Rows represent genotypes and columns represent tissues. Bold outlines indicate high significance with a *p*-value < 0.01.

The DEGs and DADMs were mapped to the common bean biological pathways in the KEGG online database. As the untargeted metabolome mainly detected the primary metabolites such as amino acids and carbohydrates, the analysis was focused on the carbon and amino acid metabolism and their connections to relevant carbohydrates (Figure 6). Comparison of the leaf tissues demonstrated the escalated feeding of citrate cycle in Ispir leaves through upregulated genes in Fructose-6P – PEP – Oxaloacetate and Fructose-6P – PEP – Pyruvate – Acetyl CoA pathways. Both malate and fumarate levels are drastically decreased in TL (*p*-value < 0.01; log_2_FC > 1). On the other hand, their levels were stable in Ispir. Carbohydrates were mainly accumulated in IL, but there is no significant change for many in TL tissues (Supplementary Figure 2). Particularly accumulation of sucrose and glucose in Ispir leaf tissues reflects the sustained carbon fixation – glycolysis cycle, which is also implied by enriched photosynthesis (Figures 2, 3A, 4).

Glutamate - Glutamine/2-Oxogluterate reaction pathway is enriched in leaf tissues of both varieties (Figure 6; **Additional File 3**). In turn, IL accumulated glutamate, TL accumulated glutamine; this indicates inequality in the direction of reaction between the genotypes. IL displayed an increased asparagine production with accumulated asparagine and an upregulated asparagine biosynthesis-related gene -Phvul. 006G069300-, while a homolog of that gene -Phvul.001G252200- was downregulated in TL (**Additional file 3**).

In the root tissues, genes related to glycolysis and citric acid cycle were more inclined to downregulation in the IR compared to TR, which displayed a more stable carbon pathway (Figure 6; **Additional File 3**). Especially downregulations on Fructose-6P – PEP – Oxaloacetate pathway genes and upregulations on Oxaloacetate to PEP and Pyruvate to PEP conversions indicated a decelerated energy metabolism in IR compared to TR (Figure 6; **Additional File 3**). While both genotypes accumulated sucrose, IR managed to accumulate fructose, mannitol, galactose, and tagatose as well (Supplementary Figure 2). In connection, the intensity difference analysis have pointed out a *sucrose-phosphate synthase* homolog Phvul.005G002600 (**Additional File 4**), that might have a role in accumulation of these carbohydrates in IR. Sucrose to glucose/fructose-6P conversions were in complex regulation in the roots of both genotypes with various up- and downregulated genes. IR accumulated glutamate but was depleted in isoleucine; TR on the other hand accumulated isoleucine, valine, threonine, glutamine, and lysine but were depleted in tryptophan in contrast to IR. Intensity difference analysis has displayed IR specific upregulation of a putative *2-oxoisovalerate dehydrogenase*, Phvul.009G132900, which is part of ‘valine, leucine and isoleucine degradation’ pathway in KEGG; increase of this enzyme in IR might have been crucial for the content difference for the related amino acids (**Additional File 4**). Both varieties accumulated proline, asparagine, aspartate, and serine amino acids. Serine appeared to be accumulated in both root tissues, yet TR had higher accumulation (log_2_FC < 1.82) compared to IR (log_2_FC < 0.94). The genes related to production of serine from fructose-6P and conversion of it to threonine/isoleucine were downregulated in IR together with a decrease in isoleucine levels. In contrast, this pathway turned out to be mainly unaffected in TR with the accumulation of serine, threonine, and isoleucine. Alternatively, IR serine accumulation might be the result of serine biosynthesis through glycolate as both IR and TR displayed a ‘*serine-glyoxylate transaminase*’ annotated gene (Phvul.006G029100) that plays role in serine production through glycolate. Yet glycolate levels were significantly decreased in IR (Figure 6; **Additional File 3**; Supplementary Figure 2).

### Ion Contents and Regulation of Ion-Transport Related Transcripts

**FIGURE 7:**
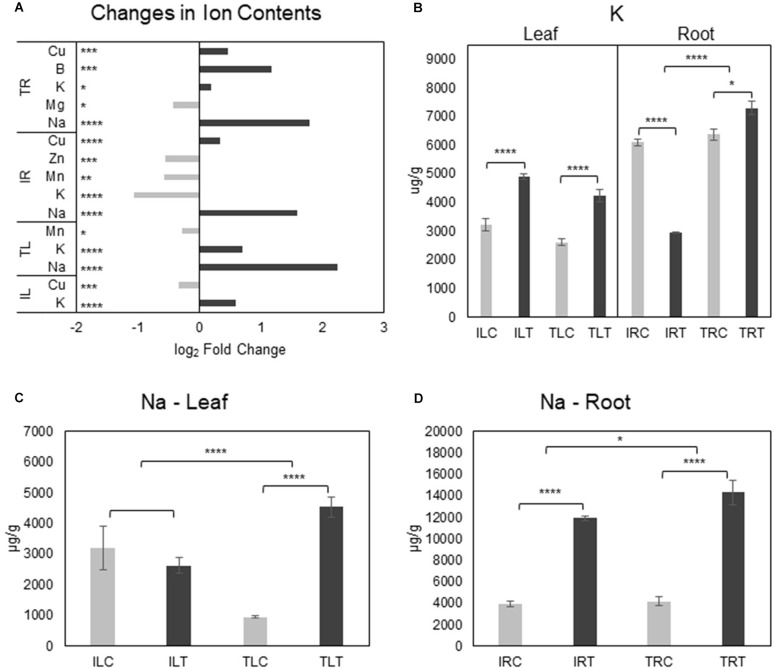
Salt responsive ion content changes for tissues and genotypes **(A)**. K^+^ **(B)** and Na^+^ **(C,D)** content changes were also displayed separately to emphasize the difference in changes between genotypes (C, control; T, salt treatment). Comparison of other ions can be found in Supplementary Figures 3, 4. * indicates significance and quantity of * displays the level of significance. (*p < 0.05; **p < 0.01; ***p < 0.005; ****p < 0.001).

Examination of tissue ion contents (**Additional File 8**) in salt-stress generated both expected and unexpected results. While the most important differences were detected in Na^+^ and K^+^ contents, there were changes in Mg^+2^, Mn^+2^, Cu^+2^, B^+3^, and Zn^+2^ contents as well (Figure 7A; Supplementary Figures 3, 4). In the root tissues of both varieties, Na^+^ ion levels were drastically increased upon salt-stress as expected, but Ispir gave a much better performance: Not only did it manage to keep the Na^+^ upsurge at significantly lower levels in the roots compared to TR43477 (Figure 7D), but also managed to keep the leaf Na^+^ levels unchanged, unlike the TR43477. Notably, Na^+^ levels were much higher in IL (3200.56 μg/g) compared to TL (955.9 μg/g) in control conditions; but in saline conditions, TL Na^+^ content drastically increased (4536.4 μg/g), while IL Na^+^ content did not demonstrate a significant change if not a decrease (2630.8 μg/g) (Figure 7C; Supplementary Figure 3). Two Na^+^/H^+^ antiporter-annotated genes were found as DEG in IR; one upregulated and one downregulated, while no Na^+^ -related transporter was differentially regulated in TR. Two Na^+^ symporter-annotated genes were uniquely found as DEGs in IL (**Additional File 3**) which might indicate their possible roles in pre-stress and stress leaf Na^+^ homeostasis in IL.

K^+^ levels significantly decreased in the salt-stress tolerant Ispir roots, while its content slightly increased in TR in salt stress conditions (Figure 6B). TR displayed four separate downregulated K^+^-transport-related DEGs; IR displayed one unique upregulated and three downregulated K^+^-transport-related DEGs. While three K^+^-transport-related DEGs were common to both IR and TR, leaves of both genotypes activated/deactivated completely different sets of genes related to the same term. However, leaf K^+^ levels were significantly boosted in both genotypes. Cu^+2^ levels dropped in IL together with five downregulated DEGs related to Cu^+2^ transport. Separately, TL Mn^+2^ content displayed a somewhat significant decrease in salinity. IL Mn + 2 content did not show a significant change upon salt stress, but in both control and treatment conditions, it was approximately 2-fold higher for IL compared to TL (Supplementary Figure 3). A similar scenario was observed for Zn^+2^ as well, with TL accumulating 3-fold higher than IL in both control and treatment conditions (Supplementary Figure 3).

Cu^+2^ levels displayed a highly significant increase in both root tissues of Ispir and TR43477 (Figure 7A; Supplementary Figure 4), but TR did not present any DEGs related to transport of this ion. In contrast, IR displayed three down- and two upregulated genes (**Additional File 3**). TR also accumulated B^+3^ and had a slight decrease of Mg^+2^. IR, on the other hand, displayed significant reductions in Mn^+2^ and Zn^+2^ contents together with mostly downregulated transporter genes related to both ions (**Additional File 3**). But Zn^+2^ levels were already significantly higher for IR in control conditions compared to TR (approx. 1.4 fold difference) (Supplementary Figure 4) and salt-treatment only reduced Zn^+2^ content of IR similar to the Zn^+2^ level in TR in treatment conditions. Interestingly, Mo^+2^ content was exceptionally higher (Supplementary Figure 4) in the tissues of Ispir in both conditions, although it did not change significantly in salt-stress condition.

## Discussion

Rise of salt content in arable lands coupled with yield drop in salt-sensitive crops has become one of the most urgent problems in the developing world. Although several effects of salinity-induced dehydration and ionic imbalance on the plant growth and development are known, many aspects of this process remain to be elucidated. Thus, it is important to decipher molecular mechanisms of salt-stress response and tolerance for the generation of salt-tolerant crops. Our study has focused on the transcriptomic, metabolomic, and ionic differences of two contrasting common bean genotypes under salinity stress to unravel the tolerance-related genes, metabolites, ions, and the pathways that connect them.

The exposure of root tissues to elevated salt levels stimulates signaling cascades that initially regulate ionic balance with Na^+^ influx restriction and root-to-shoot Na^+^ translocation. Subsequently, mechanisms that result in scavenging of toxic ions to vacuoles are activated to protect the cytoplasmic activities. The robustness of this systemic response, rather than a qualitative distinction, constitutes the main factor for the difference in tolerance between glycophytes and halophytes (Flowers and Colmer, 2008, 2015; Maathuis et al., 2014). Moreover, excessive salt in the environment results in an impasse as it both generates water stress and provide the cheap osmolytes to maintain the water potential. As this condition persists, with the accumulation of ions, escalating imbalance disrupts the molecular mechanisms in salt-susceptible species. Differential success in alleviating this stress is observed among different genotypes, which may help us understand the molecular basis and define better tolerance mechanisms.

### Carbon Fixation With Osmotic and Ionic Balance

Our ion analysis demonstrated high Na^+^ accumulation in root tissues of both genotypes upon salt stress, but IR managed to keep it at significantly lower levels than TR (Figures 7A–D). A more striking difference was observed at the leaf tissues: IL maintained the level of Na^+^ after salt treatment while there was a large-scale Na^+^ accumulation in TL. This implies a better Na^+^ exclusion ability for IR that was not due to root-to-leaf translocation of excess Na^+^ as implied by the stable IL Na^+^ levels. The much higher control condition Na^+^ level of IL compared to TL (3.3-fold, *p*-value < 0.05) (Supplementary Figure 3A) might also be an effective strategy against stress-driven Na^+^ influx to the leaves. In either case, this distinction might be one of the focal points explaining the high endurance of Ispir genotype.

On the other hand, the situation is different with K^+^ levels: The K^+^ decrease in roots of Ispir can be expected since it is a glycophyte, even though a relatively salt-tolerant genotype. The decrease in cellular K^+^ levels was observed previously both in glycophytes (Kronzucker et al., 2006; C.-M. Wang et al., 2009) and in halophytes (S. Wang et al., 2005; S.-M. Wang et al., 2007; C.-M. Wang et al., 2009). However, the roots of the susceptible TR43477 increased its K^+^ levels (Figures 7A,B). Since much of the Na^+^ entry to the root cells is through K^+^ channels (Amtmann and Sanders, 1998; Laurie et al., 2002; Garciadeblás et al., 2003; R. A. James et al., 2011), it is possible that Ispir roots prevent the excessive Na^+^ influx via closure of K^+^ channels, which as a trade-off, may result in lower K^+^ levels. A less successful approach adopted by TR43477 evidently allows for high Na^+^ accumulation both in leaf and in root tissues together with K^+^. Potentially better vacuole sequestration in roots on top of better exclusion ability might mitigate the negative effect of slightly lower K^+^/Na^+^ ratio in the Ispir roots. Another important aspect of this distinction might be the ability to achieve a much favorable K^+^/Na^+^ ratio in the leaves of the Ispir genotype. IL has managed to increase its K^+^/Na^+^ ratio by 1.8-fold while the same ratio decreased by 2.9-fold in TL under salt stress. This asymmetry, on the other hand, could be a vital element for the observed contrast in photosynthetic capacities of these genotypes.

Salt stress builds a major constraint on the photosynthetic capacity (Munns et al., 2006). Na^+^ accumulation in chloroplasts affects growth mainly by disrupting the photosynthetic electron transport (Boyer, 1976; Kirst, 1990) and inhibiting the PSII activity (Mishra et al., 1991; Everard et al., 1994; Kao et al., 2003; Parida et al., 2003). Moreover, it decreases chlorophyll content in susceptible plants such as potato (Abdullah and Ahmad, 1990), tomato (Moghaieb et al., 2001), pea (Hamada and El-Enany, 1994), as well as common bean (Seemann and Critchley, 1985). However, Ispir, unlike TR43477, displayed a boosted carbon fixation metabolism with enriched GO terms and KEGG pathways (Figures 2, 3) and an active chlorophyll content regulation (Figure 4A). Photosynthesis capacity is firmly connected to stomata, which control water loss/photosynthesis balance (Jones, 1998). As a major stomatal guard cell osmoregulator (Talbott and Zeiger, 1996; Hedrich, 2012), K^+^ coordinates the gas exchange and transpiration rates (Kim et al., 2010), which can be heavily affected by salt-stress (Liu et al., 2017). Accumulated Na^+^ competes with K^+^ for the regulation of stomata, which causes considerable side effects such as deregulation through ABA and CO_2_ (Jaschke et al., 1997; Fricke et al., 2004). Since salt-tolerant plants are known to have more efficient regulation of stomata compared to sensitive ones (Hedrich and Shabala, 2018), superior stomatal regulation through a higher K^+^/Na^+^ ratio for Ispir leaves may be the essence of its enhanced photosynthetic capacity. This connection is further implied by the decreased glycolate production in Ispir leaf tissues, which is an indication of reduced photorespiration, a metabolism that consumes ATP together with the reducing power of the photosynthetic electron transport system and reduces the efficiency of CO_2_ fixation (Keys et al., 1986). As photosynthesis/photorespiration rate depends mainly on the CO_2_/O_2_ levels which on the other hand depends on stomatal density and conductance (Hetherington and Woodward, 2003; Chen et al., 2017; Zhao et al., 2019), Ispir potentially is a better stomatal regulator compared to TR43477 under salt stress. An enrichment of pectinesterase related terms and genes in Ispir leaves (Figure 2A, **Additional File 3**) further supports this hypothesis: Amsbury et al. (2016) have demonstrated that the guard cells in Arabidopsis have high content of un-esterified pectins. The guard-cell pectins were shown to be esterified in an Arabidopsis mutant deficient for a potent *pectinesterase* (*PME6*). The lack of this enzyme resulted in a decreased guard cell dynamic motility and, in turn, crippled the stomatal function causing susceptibility to low-water conditions. Indeed, one of the pectinesterase genes (Phvul.001G209400) that displayed an upregulation in IL is a somewhat close homolog (%43 similarity) of Arabidopsis *PME6;* thus, might be playing a similar role in common bean. A decreased glycolate content and enriched pectinesterase-related terms along with the enriched photosynthesis pathway genes, increased chlorophyll content, and highly upregulated genes that were annotated as *Rubisco* (**Additional File 3**), are good indicators of an increased photosynthesis/lower photorespiration rate and a better stomatal regulatory system in Ispir genotype.

The improved photosynthetic capacity and enriched carbon fixation (Figures 2A, 3A, 4) in IL have conceivably yielded accumulation of various carbohydrates (nine types including sucrose and glucose- Figure 5) and enrichment in sucrose and starch metabolism (Figure 3A). Notably, intense upregulation of a *starch synthase* (Phvul.004G029100) together with a β-*amylase* (Phvul.011G107700), which is involved in starch breakdown (Scheidig et al., 2002), in IL also implied an enriched starch metabolism (**Additional File 4**). In turn, TL was depleted in such metabolites and exhibited a decrease in polysaccharide metabolic processes (Figure 2A). The augmented carbohydrate metabolism and soluble sugar contents in Ispir point to a superior tolerance mechanism in this genotype as carbohydrates provide osmoprotection, membrane stability, and turgor maintenance under osmotic stress (Ende and Peshev, 2013). Soluble carbohydrates are also known to be a metabolic source of energy, act as signaling molecules in plant growth regulation (Jang and Sheen, 1994; Pego et al., 2000) and to have roles in plant-stress response regulation (Ho et al., 2001; Chinnusamy et al., 2004). The variation in the resulting carbohydrate quantity might also explain the difference between the carbohydrate levels of the root systems (Figure 5; Supplementary Figure 2). Specifically, accumulation of mannitol (log_2_FC – 2.76; *p*-value < 0.01), which is a well-known osmoprotectant (Tarczynski et al., 1993; Thomas et al., 1995; Shen et al., 1997; Abebe et al., 2003) and hydroxyl radical quencher (Smirnoff and Cumbes, 1989; Shen et al., 1997) may be important for the tolerance of Ispir roots to the excessive ion uptake (Stoop et al., 1996) and osmoregulation (Hellebusi, 1976).

Another implication of ionic balance regulation difference between the roots of these two genotypes is the significant reductions in IR Mn^+2^ and Zn^+2^ levels (Figure 7A). Especially Zn^+2^ was demonstrated to have positive effects on abiotic stress tolerance (Bagci et al., 2007; Ahmad et al., 2017). In our study, salt treatment caused a significant reduction of Zn^+2^ level in IR (from 1674.8 μg/g to 1143.4 μg/g). In TR, however, the control-condition level was already low (1.3-fold compared to IR, *p*-value < 0.005) and remained almost unchanged after the exposure to salt stress (a decrease from 1214.12 μg/g to 1184.2 μg/g) (Supplementary Figures 4C,D). It is intriguing to further explore this difference to understand if the higher initial content of Zn^+2^ contributes to the salt tolerance. One possibility is that Zn^+2^ normally accumulated in IR was incorporated into Zn-containing metalloproteins (McCall et al., 2000) required for an efficient response such as *alcohol dehydrogenase* (Shi et al., 2017), *carbonic anhydrase* (Yu et al., 2007), and *superoxide dismutase* (Bowler et al., 1992). As TR roots did not have such a pool of Zn^+2^ they might have failed to address the stress condition as efficiently as IR due to the lower activity/availability of such metalloproteins. On the other hand, salt-treatment caused Mn^+2^ decrease in IR may be due to allocation of the ion to the leaf tissues for protection of relatively high leaf Mn^+2^ content in Ispir (1.9-fold higher in control conditions compared to TL – Supplementary Figures 3C,D) while already low TL Mn^+2^ content displayed a significant reduction in treatment conditions. Since Mn^+2^ is an essential element for photosystem II to function and its scarcity disrupts the photosynthetic efficiency and stability (Gavalas and Clark, 1971; Husted et al., 2009; Schmidt et al., 2015), this allocation pattern might be a crucial aspect of tolerance as well.

However, together with significant reductions in Mn^+2^ and K^+^ contents (while only Mg^+2^ content displayed a slightly significant decrease in TR43477), Ispir roots displayed a distinct pattern (Figure 7A). A pattern that might also suggest regulation of ionic balance by lowering the concentration of necessary cations in the root system to a bare minimum to counteract the effects of toxic levels of Na^+^ ions. Evidently, Ispir genotype manages to not only preserve, if not boost, its energy metabolism but also to increase its photosynthesis intensity, which is a basic tolerance mechanism in low-water conditions (Santos and Pimentel, 2009; Loutfy et al., 2012). This is most probably feasible for the Ispir leaves as the stable Na^+^ level throughout stress did not affect any enzymatic activity and did not disrupt the osmotic and ionic balance.

### Protein and Amino Acid Metabolism

Natural variation in salinity-tolerance is very high in the plant kingdom, even within the same species, which is reflected in different growth responses of different genotypes (Munns and Tester, 2008). Under the condition of salt-stress, IL displayed diminished protein production and modification but enriched protein protection-related terms (Figure 2). It is possible that keeping translation to a minimum is an efficient way to protect the proteome from oxidative stress associated with salinity conditions. On the other hand, the response of TL was less conservative: Terms for transcription were increased but translation and proteolysis terms were decreased (Figure 2A). The allocation of energy and valuable elements to transcription under conditions where normal levels of translation are harmful or not possible may be one of the weak points of the salt-sensitive genotype. Root systems were different in their response too: IR displayed a reduction in growth and production-related terms, while TR was only depleted in nitrogen metabolism (Figure 3B). Keeping the root smaller under salinity conditions may be one of the ways to limit the exposure of the nutrient-uptake interface to saline environment. Curiously, TR accumulated the highest number of amino acids among tissues and genotypes (valine, isoleucine and lysine were unique to TR). Although accumulation of amino acids is generally assumed as representation of tolerance (see review (Batista-Silva et al., 2019), catabolism of lysine, valine, isoleucine, and leucine were recognized as important pathways for osmotic stress tolerance in Arabidopsis (Pires et al., 2016). Pointedly, IR did not accumulate valine and had decreased amount of isoleucine in response to salt stress (Figures 5C, 6) and this might be due to upregulation of a *2-oxoisovalerate dehydrogenase* homolog, Phvul.009G132900 (**Additional File 4**), which was demonstrated to have a major role in branched-chain amino acid catabolism (Fujiki et al., 2002). Increase in lysine content in TR deserves special attention, since, as mentioned above, it may be considered as a tolerance mechanism as lysine accumulates in some drought-tolerant plant ecotypes (Yadav et al., 2019; You et al., 2019). But lysine catabolism, especially SACPATH pathway, a highly stress-responsive protective system (Markovitz and Chuang, 1987; Arruda et al., 2000; Azevedo et al., 2003; Moulin et al., 2006; Kiyota et al., 2015; Michaletti et al., 2018; Yadav et al., 2019; You et al., 2019), appeared to be inactive in the TR43477 compared to Ispir: The latter genotype has two upregulated SACPATH pathway genes in the genome (**Additional File 3**) including the only gene annotated as *lysine-ketoglutarate reductase/saccharopine dehydrogenase*. SACPATH pathway can lead to production of proline via glutamate or α-aminoadipate (Lawrence and Grant, 1964; Sodek and Wilson, 1970; Brandt, 1975). Although the proline levels were increased in both tissues and genotypes, glutamate was only accumulated in Ispir tissues (Figures 5C, 6; Supplementary Figure 2). Thus the elevated lysine level in TR may be simply the result of more intensive proteolysis associated with stress (for a review, see Hildebrandt et al., 2015).

The difference in glutamate/glutamine biosynthesis also deserves attention as glutamate-glutamine/2-oxogluterate reaction is enriched in both varieties but glutamate/glutamine conversion pathway is mainly activated in Ispir tissues (Figure 6; **Additional File 3**). While both tissues of Ispir accumulated glutamate, TR43477 tissues accumulated glutamine, which indicates an inequality in the reaction direction for these varieties. Glutamate is essential for stress tolerance as it was demonstrated to support amino-acid synthesis under osmotic stress (Ramos et al., 2005), activate stress tolerance pathways via H_2_O_2_ burst (Lei et al., 2017), act as a signaling molecule for stress response pathways (Kan et al., 2017) and regulate the stomatal aperture under low-water conditions (Yoshida et al., 2016; Qiu et al., 2020). Glutamate is also necessary for biosynthesis of glutathione, an active compound of antioxidant defense system (Lu, 2013). Glutamine has also been implicated in stress-responses, acting as a regulator of a transcription factor (Kan et al., 2015). Overexpression of the enzyme necessary for its production, glutamine synthase, yields better abiotic stress tolerance in several species (Lee et al., 2013; D. James et al., 2018). Still, glutamate appears to be a hub for stress response patterns.

Moreover, the imbalance of regulation in glutamate/glutamine cycle genes (Figure 6; **Additional File 3**) may be another indication of the difference in stress-responsive nitrogen metabolism for these genotypes (Masclaux-Daubresse et al., 2006; Zhang et al., 2017) as implied by the KEGG pathway analysis (Figures 3A,B). Concerning that, asparagine biosynthesis and content were also differentially regulated between genotypes and tissues. Asp was mainly accumulated in the root tissues of both genotypes, but also displayed low but significant accumulation in the leaves of Ispir (Figures 5C, 6; Supplementary Figure 2). An “*asparagine synthase-1*” annotated gene (Phvul.006G069300) was upregulated in all tissues except TL, where a different member of this gene family annotated as “*asparagine synthase-3*” (Phvul.001G252200) was downregulated. If the elevated Asp levels in TR were high mainly due to increased proteolysis as discussed above, the high accumulation of this amino acid in both tissues of Ispir may indicate the better nitrogen storage capacity of this genotype, since asparagine is known to be a good nitrogen reserve molecule (Pate, 1980; Lea et al., 2007). As salinity declines nitrogen assimilation and acquisition capacity of plants (Gouia et al., 1994; Debouba et al., 2006), and nitrogen is a necessary building block for amino acids, hormones such as auxin and other important amine-compounds, nitrogen withholding may be another key aspect of Ispir’s salt tolerance.

### Other Aspects of Tolerance

Certainly, salt tolerance is a complex feature that cannot be attributed to only a few biological processes such as carbon fixation and amino acid biosynthesis of primary metabolism (Munns and Tester, 2008; Gupta and Huang, 2014). Secondary metabolism is also known to be highly responsive to environmental factors including salt stress (Gupta and Huang, 2014; Sytar et al., 2018). Our previous study on Ispir in salt stress has displayed the enrichment of secondary metabolism genes in response to salt in both leaves and roots (Hiz et al., 2014). The present comparative study demonstrated that the roots of Ispir were differentially enriched in KEGG terms related to terpenoid metabolism (Figure 3B), a type of metabolism that involves volatile unsaturated hydrocarbon compounds with high structural diversity (Degenhardt et al., 2009). Other studies have reported terpenoids to upsurge in response to saline conditions and to be involved in tolerance responses (Harborne, 1999; Bourgaud et al., 2001; Sytar et al., 2018). A recent study in maize demonstrated the accumulation of phytoalexin terpenoids in roots as a key feature of an abiotic stress response and hormonal regulation under stress conditions (Vaughan et al., 2015). Terpenoid biosynthesis has been associated with photosynthetic machinery, especially chloroplasts, in non-stressed plants before (Loreto et al., 1996; Sharkey et al., 1996) and the impairment of terpenoid production in drought stress conditions was related to a decrease in levels of available substrates due to disrupted photosynthesis (Peñuelas et al., 2009; Šimpraga et al., 2011; Kleine and Müller, 2014; Nogués et al., 2015). The photosynthetic machinery in Ispir, compared to TR43477, displayed a rather boosted response in salt-stress conditions, thus enabling the production of new terpenoids in the roots. Moreover, IR was diminished in esterase and alcohol catabolism-related GO terms (Figure 2B), which indicates improved conservation of secondary metabolites such as terpenoids (Isah, 2019).

The prevention of salinity-induced reduction of photosynthetic activity in Ispir might also had a positive outcome for respiratory metabolism. Since salt stress does not affect cellular O_2_ levels, the respiration rate mainly depends on the supply of substrate and biochemical regulation. Thus, the negative effect of salt stress on the respiratory machinery can be attributed to lower carbon fixation (Seemann and Critchley, 1985; Centritto et al., 2003) and disruption of electron transport chain due to high accumulation of ions (T. J. Flowers, 1974). Although there is no concrete evidence for the effect of increased or decreased respiration rates on salt-tolerance (Jacoby et al., 2011), respiratory homeostasis was correlated with better tolerance responses in a few species (Kasai et al., 1998; Jacoby et al., 2011). In this regard, the elevated levels of lactic acid in TL (log_2_FC – 0.97; *p*-value < 0.01) (Figure 5C; Supplementary Figure 2) might be an indicator of perturbed mitochondrial and increased anaerobic respiration. In contrast, lactic acid levels in IL have slightly decreased in response to salt stress (log_2_FC – −0.64; *p*-value < 0.05), which might also be related to higher intensity of alcohol metabolism in IL. Out of five homologs of *alcohol dehydrogenase 1* (*ADH1*), one (Phvul.001G067300) was significantly upregulated in IL, while the others were not regulated in either genotype. The activity of this gene might have been sufficient for the reduction of toxic acetaldehyde (Bondy, 1992; M. Zhang et al., 1997) to ethanol, thus preventing the accumulation of lactic acid in IL. Additionally, *ADH1* upregulation might have had other positive effects since this gene is known to respond to abiotic-stresses and is essential for tolerance to osmotic and salt stresses (Conley et al., 1999; Shi et al., 2017; Yi et al., 2017).

Like other cellular activities, lipid metabolism is also affected by salt stress (Parida and Das, 2005). Indeed, in our study, the lipid content was low in TR43477 tissues (Figure 5C), which may be an indicator of susceptibility, as reported before in drought stress conditions. (Yordanov et al., 2000; Alpaslan et al., 2001).

Finally, besides the salt-caused differences, the constitutive 55- to 177-fold difference in Mo content between Ispir and TR43477 tissues under both conditions (Supplementary Figures 3E,F, 4E,F) can be important for salt tolerance in Ispir. Mo has been reported in many studies to improve abiotic stress tolerance in drought, salinity and low temperature conditions (Sun et al., 2009, 2014; Zhang et al., 2012; Wu et al., 2014). In saline conditions, Mo was demonstrated to regulate the antioxidant machinery and osmotic balance in Chinese cabbage (Zhang et al., 2012). Furthermore, it was reported to increase chlorophyll and carotene contents together with photosynthesis rate and have a positive effect on ionic balance regulation in the same species (Zhang et al., 2014). Thus, it is conceivable that the higher accumulation of Mo in Ispir contributes to its superior salt tolerance.

## Conclusion

To understand the molecular basis of differential response to salt stress in two common bean genotypes, we performed comprehensive analyses of transcript levels, abundance of different metabolites, and ionic content in the root and leaf tissues of these genotypes. Our data suggest that the preservation of photosynthetic machinery via the control of Na^+^ accumulation in leaves and the efficient sequestration of K^+^ in roots may be vital for the stability of carbohydrate and energy metabolisms under saline conditions. Together with the resulting osmoprotection and higher substrate availability, a better regulation of amino acid metabolism, the remarkable shift in the ratio between glutamine and glutamate, the maintenance of ionic balance and the higher accumulation capacity for certain ions, such as Mo and Mn in roots and leaves and Zn in roots, might be the fundamentals of salt-tolerance in Ispir genotype of common bean. Functional studies on candidate genes and pathways highlighted in this study will improve our understanding of salt-tolerance and facilitate the generation of salt-tolerant plants.

## Data Availability Statement

High-throughput sequencing data generated in this study have been deposited to the NCBI GEO Datasets and can be accessed by GEO accession number: GSE156113.

## Author Contributions

HN and MT conceived and designed the experiment. HN conducted plant growth, stress treatments, RNA isolation, and metabolite extraction. mRNA library preparation and RNA-Seq was conducted by Macrogen, Inc. (S. Korea). HN performed the RNA-Seq raw data analysis. BS and NB performed the GC-MS measurements, data collection and metabolite identification. ICP-MS and ICP-OES measurements were conducted by Yildiz Technical University Merklab. (Turkey/Istanbul). Statistical and bioinformatics analyses for transcriptome, metabolome and ionome data were performed by HN. HN and MT wrote the manuscript with valuable contributions from all authors.

## Conflict of Interest

The authors declare that the research was conducted in the absence of any commercial or financial relationships that could be construed as a potential conflict of interest.

## Footnotes

1 bioinformatics.babraham.ac.uk/projects/seqmonk/

## Supplementary Material

Supplementary Figure 1 qRT-PCR verification of RNA-Seq Data. RNAseq and qPCR results (log_2_ fold change (FC)) of 10 genes from Ispir **(A)** and TR43477 **(B)** root tissues were compared to each other. “r” depicts Pearson’s r.

Supplementary Figure 2 Graph representation of significantly accumulated and decreased metabolites in saline conditions. Highly significant (*p*-value < 0.01) changes were pointed out with asterisk. **p* < 0.05; ***p* < 0.01; ****p* < 0.005; *****p* < 0.001.

Supplementary Figure 3 Comparison of leaf ion contents in control and saline conditions. **(A,B)** Display the macroelement content comparisons for control and treatment conditions respectively. **(C–F)** Display the microelement content comparisosn for control and treatment conditions respectively. **p* < 0.05; ***p* < 0.01; ****p* < 0.005; *****p* < 0.001.

Supplementary Figure 4 Comparison of root ion contents in control and saline conditions. **(A,B)** Display the macroelement content comparisons for control and treatment conditions respectively. **(C–F)** Display the microelement content comparisosn for control and treatment conditions respectively. **p* < 0.05; ***p* < 0.01; ****p* < 0.005; *****p* < 0.001.
