# Corrigendum: Comparative Transcriptome, Metabolome, and Ionome Analysis of Two Contrasting Common Bean Genotypes in Saline Conditions

**DOI:** 10.3389/fpls.2021.711806

**Published:** 2021-06-29

**Authors:** Harun Niron, Nazire Barlas, Bekir Salih, Müge Türet

**Affiliations:** ^1^Department of Molecular Biology and Genetics, Bogazici University, Istanbul, Turkey; ^2^Department of Chemistry, Hacettepe University, Ankara, Turkey

**Keywords:** common bean, metabolome, *Phaseolus vulgaris* L., salt-stress, tolerance, transcriptome

In the original article there were mistakes in **Figure 7**, **Supplementary Figure 4**, and **Additional File 8** as published.

The numbers defining the amount of sodium ion (Na^+^) in the root tissues of salt treated plants were erroneous by a factor of 10. The corrected Figure 7, Supplementary Figure 4, and Additional File 8 appear.

**Figure 7:**
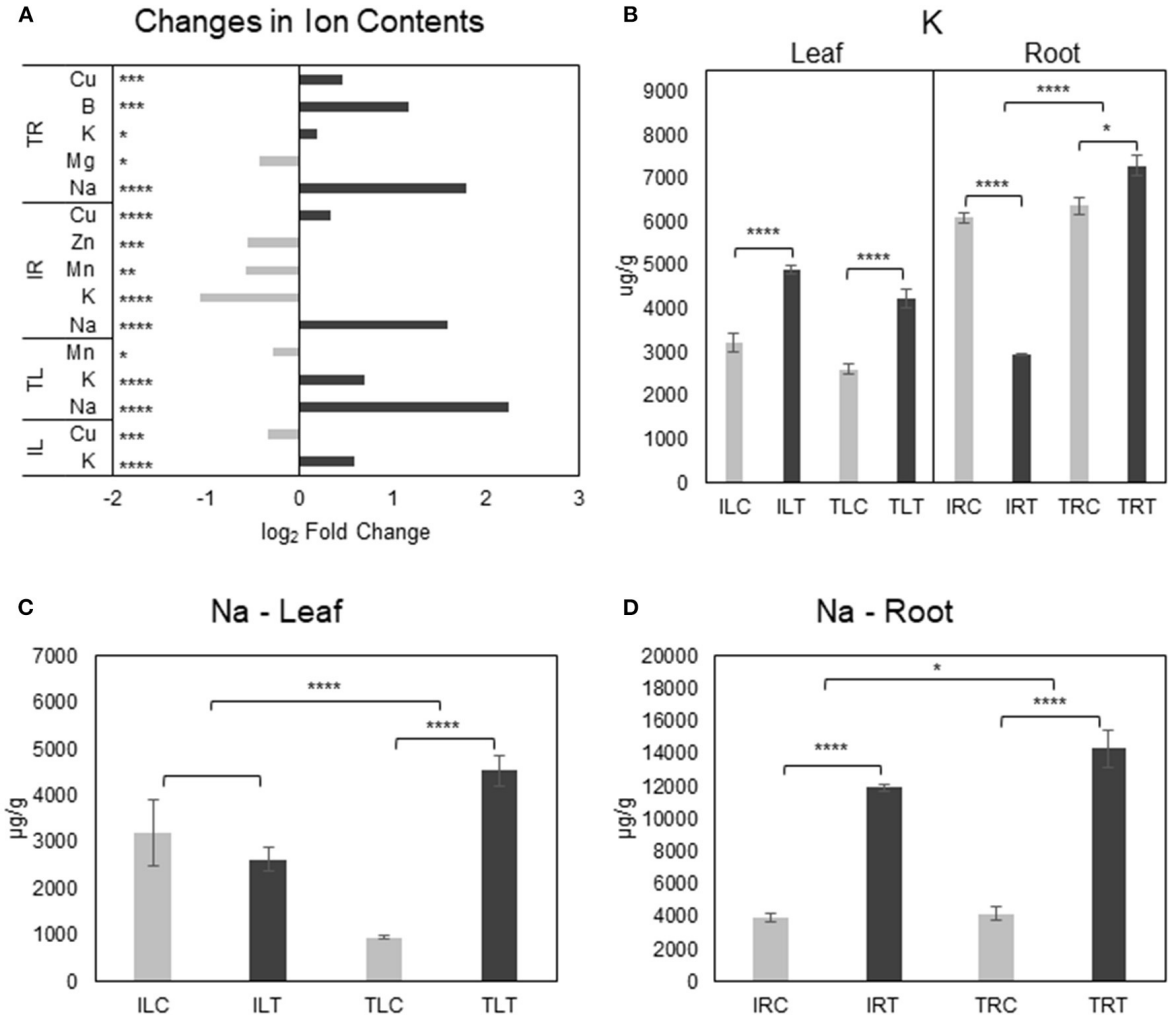
Salt responsive ion content changes for tissues and genotypes **(A)**. K^+^ **(B)** and Na^+^ **(C,D)** content changes were also displayed separately to emphasize the difference in changes between genotypes (C, control; T, salt treatment). Comparison of other ions can be found in **Supplementary Figures 3**, 4. ^*^ indicates significance and quantity of ^*^ displays the level of significance. (^*^p < 0.05; ^**^p < 0.01; ^***^p < 0.005; ^****^p < 0.001).

**Supplementary Figure 4:**
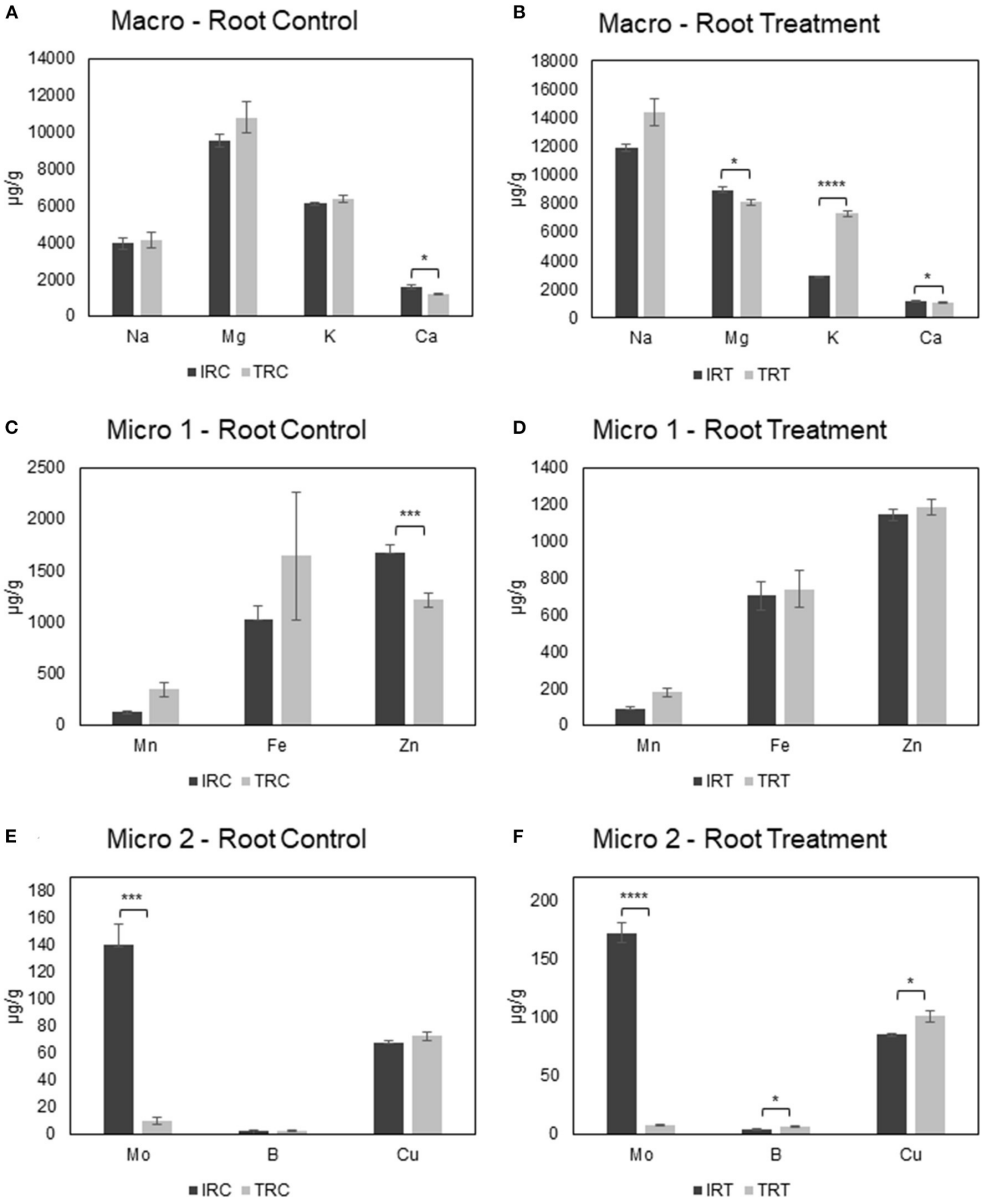
Comparison of root ion contents in control and saline conditions. **(A,B)** Display the macroelement content comparisons for control and treatment conditions respectively. **(C–F)** Display the microelement content comparisosn for control and treatment conditions respectively. ^*^p < 0.05; ^**^p < 0.01; ^***^p < 0.005; ^****^p < 0.001.

**Additional File 8:** 

**Tissue/Ion**	**ILC**		**ILT**		**TLC**		**TLT**		***p*****-value (Condition or Genotype)**			
	**Average (μg/g)**	**St. Err**.	**Average (μg/g)**	**St. Err**.	**Average (μg/g)**	**St. Err**.	**Average (μg/g)**	**St.Err**.	**ILC vs. ILT**	**TLC vs. TLT**	**ILC vs. TLC**	**ILT vs. TLT**
Na	3,200.56	711.50	2,630.80	266.42	955.90	43.95	4,536.40	318.10	0.531548	0.000470	0.047587	0.003630
Mg	6,912.60	423.75	6,978.20	139.16	6,744.60	294.62	6,403.20	176.21	0.900617	0.405283	0.779220	0.052927
K	3,220.40	198.52	4,895.20	99.95	2,604.20	113.28	4,222.00	213.25	0.000556	0.000919	0.050198	0.045284
Ca	6,465.80	291.40	6,431.40	148.82	7,287.40	243.28	6,982.20	120.98	0.928167	0.354681	0.090074	0.034319
Mn	251.24	23.74	230.00	11.64	130.06	3.91	107.81	5.06	0.500350	0.015596	0.009546	0.000223
Fe	115.99	12.89	83.34	14.14	103.94	13.12	85.26	8.73	0.165834	0.324456	0.574183	0.920976
Zn	82.91	7.74	81.96	3.85	258.82	8.08	266.30	12.24	0.925291	0.662294	0.000001	0.000069
Mo	13.11	1.50	10.42	0.43	1.21	0.21	1.24	0.13	0.186713	0.910741	0.001858	0.000013
B	33.92	4.32	26.68	0.83	47.10	4.25	38.91	3.41	0.210658	0.217989	0.087716	0.030703
Cu	8.44	0.30	6.67	0.12	9.58	0.92	7.04	0.20	0.003961	0.067807	0.340778	0.210593
**Tissue/Ion**	**IRC**		**IRT**		**TRC**		**TRT**		***p*****-value (Condition or Genotype)**			
	**Average (μg/g)**	**St. Err**.	**Average (μg/g)**	**St. Err**.	**Average (μg/g)**	**St. Err**.	**Average (μg/g)**	**St.Err**.	**IRC vs. IRT**	**TRC vs. TRT**	**IRC vs. TRC**	**IRT vs. TRT**
Na	3,933.20	286.98	1,1902.00	213.90	4,146.00	422.22	1,4354.76	941.21	0.0000001	0.000137	0.720240	0.079241
Mg	9,533.00	373.79	8,969.80	217.32	1,0781.60	854.08	8,088.40	187.21	0.285407	0.046213	0.280206	0.025641
K	6,098.80	108.89	2,937.60	23.38	6,368.60	202.81	7,293.00	222.85	0.000007	0.025503	0.334053	0.000055
Ca	1,548.20	116.70	1,195.00	27.86	1,173.40	42.95	1,089.40	24.16	0.052008	0.175876	0.042440	0.034105
Mn	133.94	7.97	90.64	5.61	344.50	68.25	177.41	24.89	0.005084	0.094286	0.050369	0.033796
Fe	1,031.80	127.32	704.52	77.29	1,644.96	619.72	740.70	98.48	0.092639	0.263811	0.431357	0.802912
Zn	1,674.80	80.12	1,143.40	31.28	1,214.12	64.93	1,184.20	44.42	0.002360	0.743602	0.004328	0.522796
Mo	140.04	15.41	171.90	8.35	10.14	2.53	7.63	1.02	0.153725	0.446274	0.001412	0.000051
B	2.68	0.50	3.81	0.51	2.74	0.17	6.22	0.52	0.191954	0.002626	0.920176	0.018254
Cu	67.14	1.55	85.11	0.89	72.44	3.28	100.76	4.56	0.000076	0.002522	0.241887	0.035853

The authors apologize for this error and state that this does not change the scientific conclusions of the article in any way. The original article has been updated.

