# Supplementary figures and images for: Comparative Transcriptome, Metabolome, and Ionome Analysis of Two Contrasting Common Bean Genotypes in Saline Conditions
Source: Front Plant Sci. 2020 Dec 10;11:599501. doi: 10.3389/fpls.2020.599501 (PMC7758407; doi:10.3389/fpls.2020.599501)

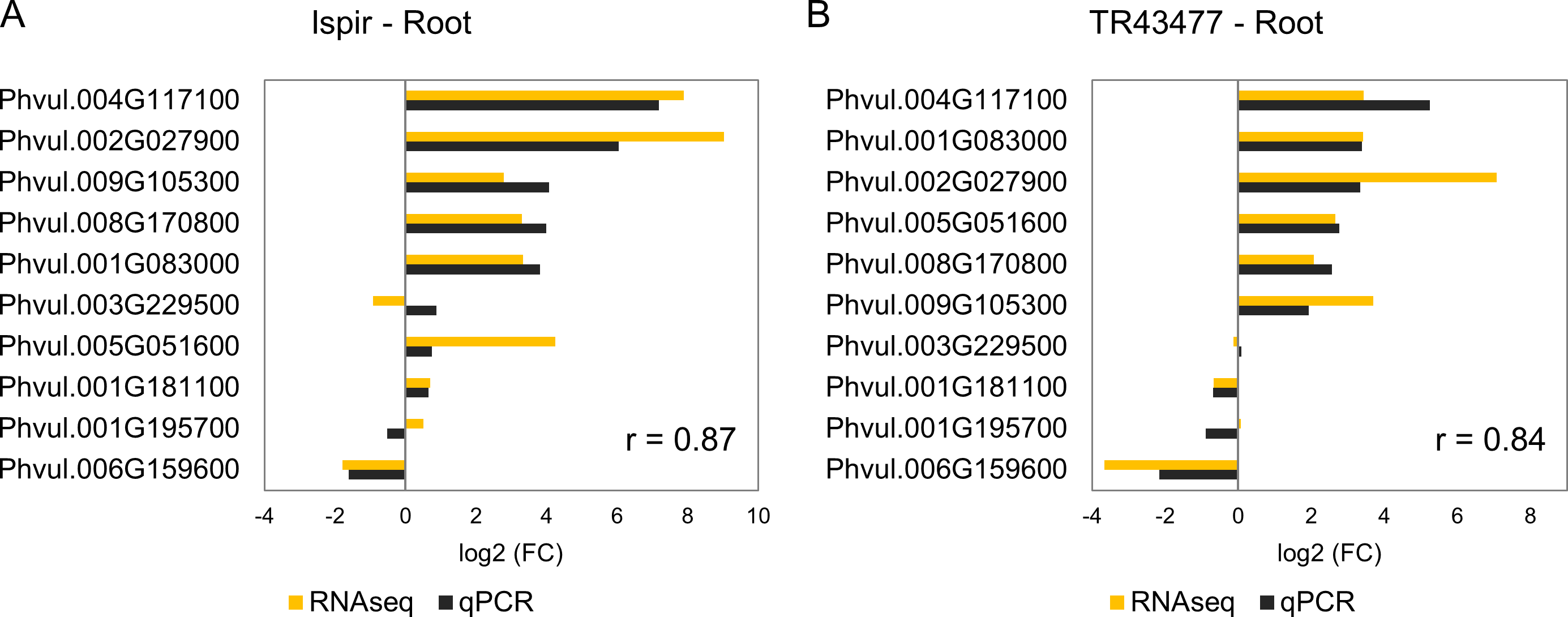

Supplement: Supplementary Figure 1 — qRT-PCR verification of RNA-Seq Data. RNAseq and qPCR results (log2 fold change (FC)) of 10 genes from Ispir (A) and TR43477 (B) root tissues were compared to each other. “r” depicts Pearson’s r. [file Image_1.TIF]

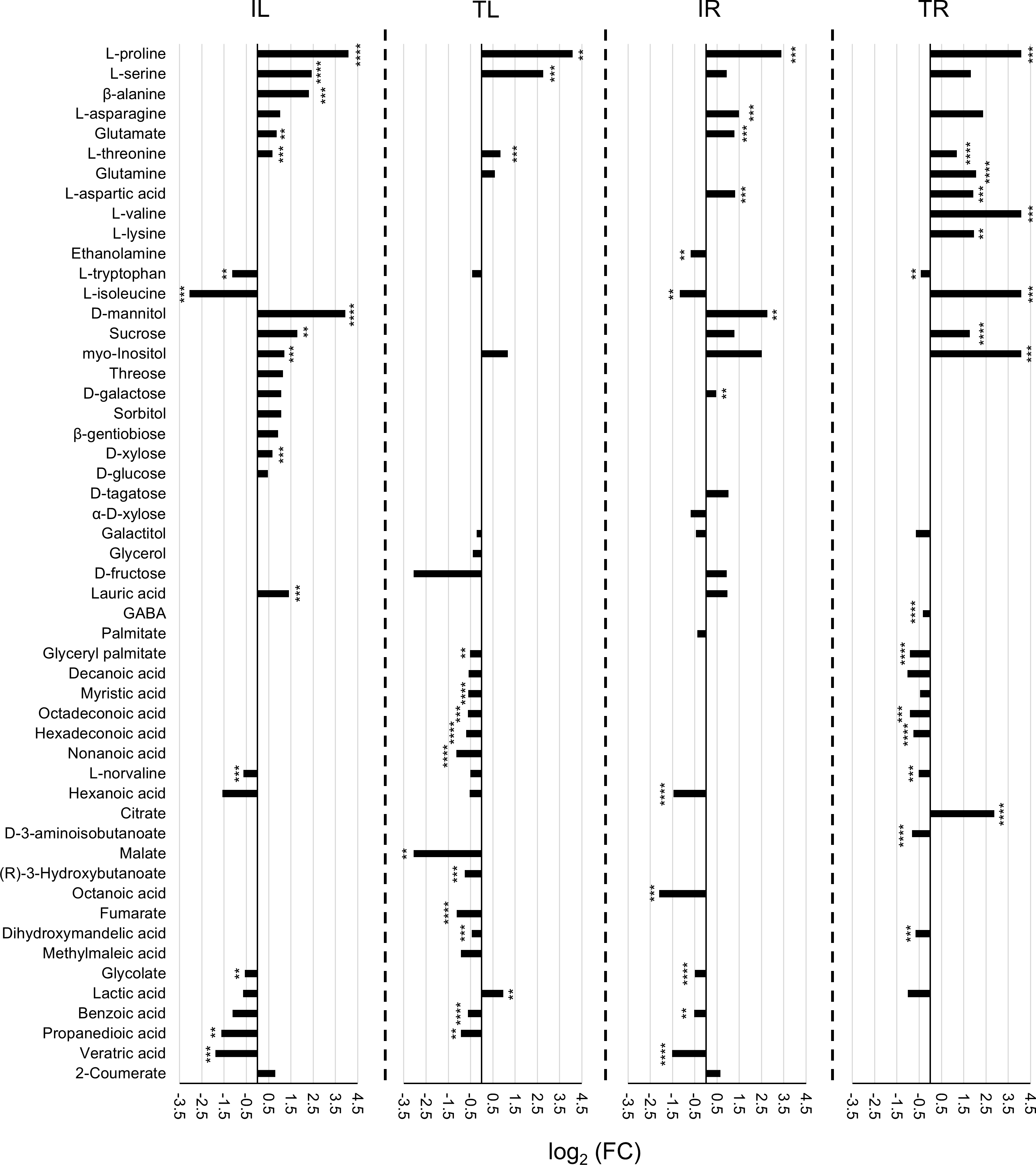

Supplement: Supplementary Figure 2 — Graph representation of significantly accumulated and decreased metabolites in saline conditions. Highly significant (p-value < 0.01) changes were pointed out with asterisk. *p < 0.05; **p < 0.01; ***p < 0.005; ****p < 0.001. [file Image_2.TIF]

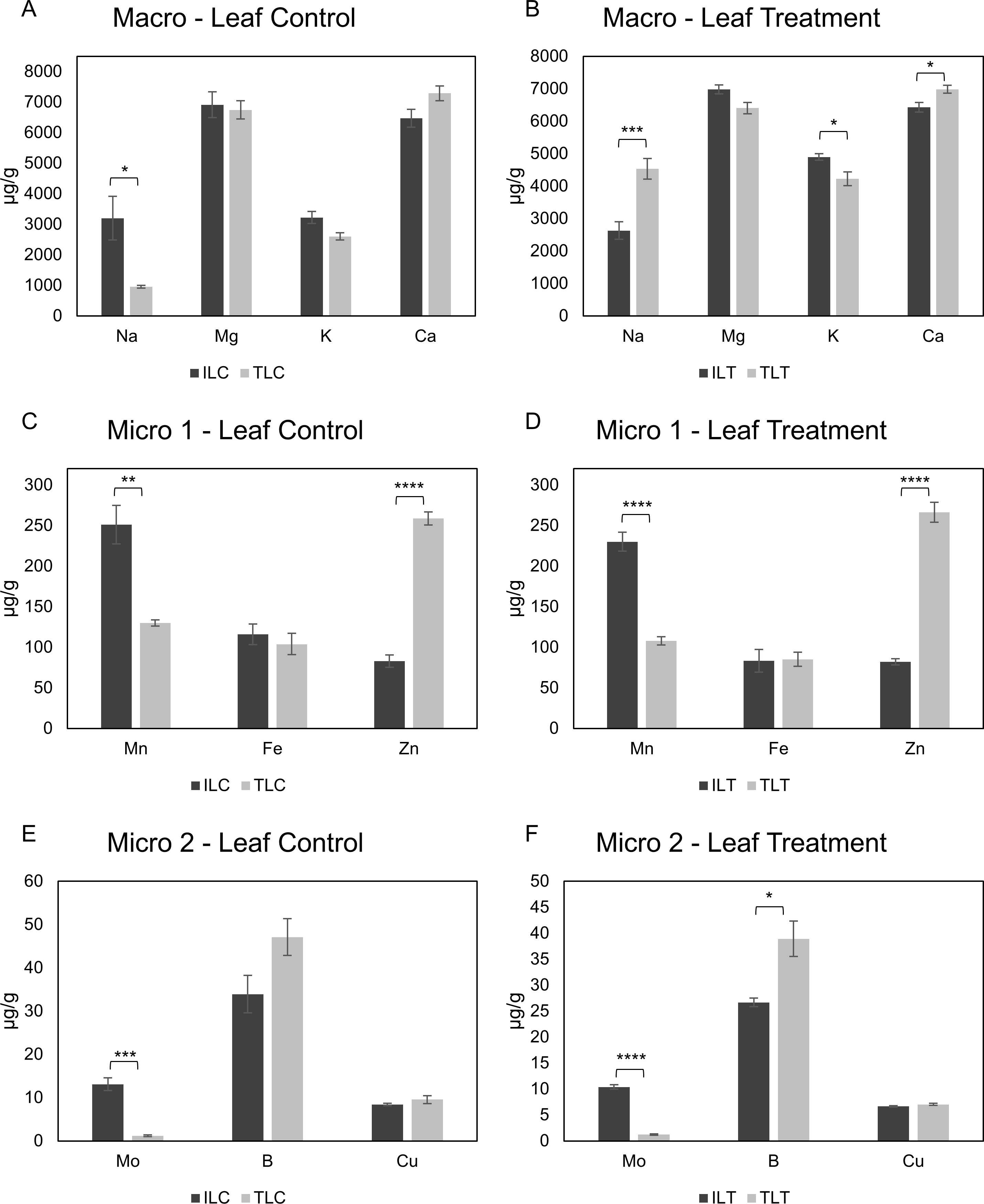

Supplement: Supplementary Figure 3 — Comparison of leaf ion contents in control and saline conditions. (A,B) Display the macroelement content comparisons for control and treatment conditions respectively. (C–F) Display the microelement content comparisosn for control and treatment conditions respectively. *p < 0.05; **p < 0.01; ***p < 0.005; ****p < 0.001. [file Image_3.TIF]

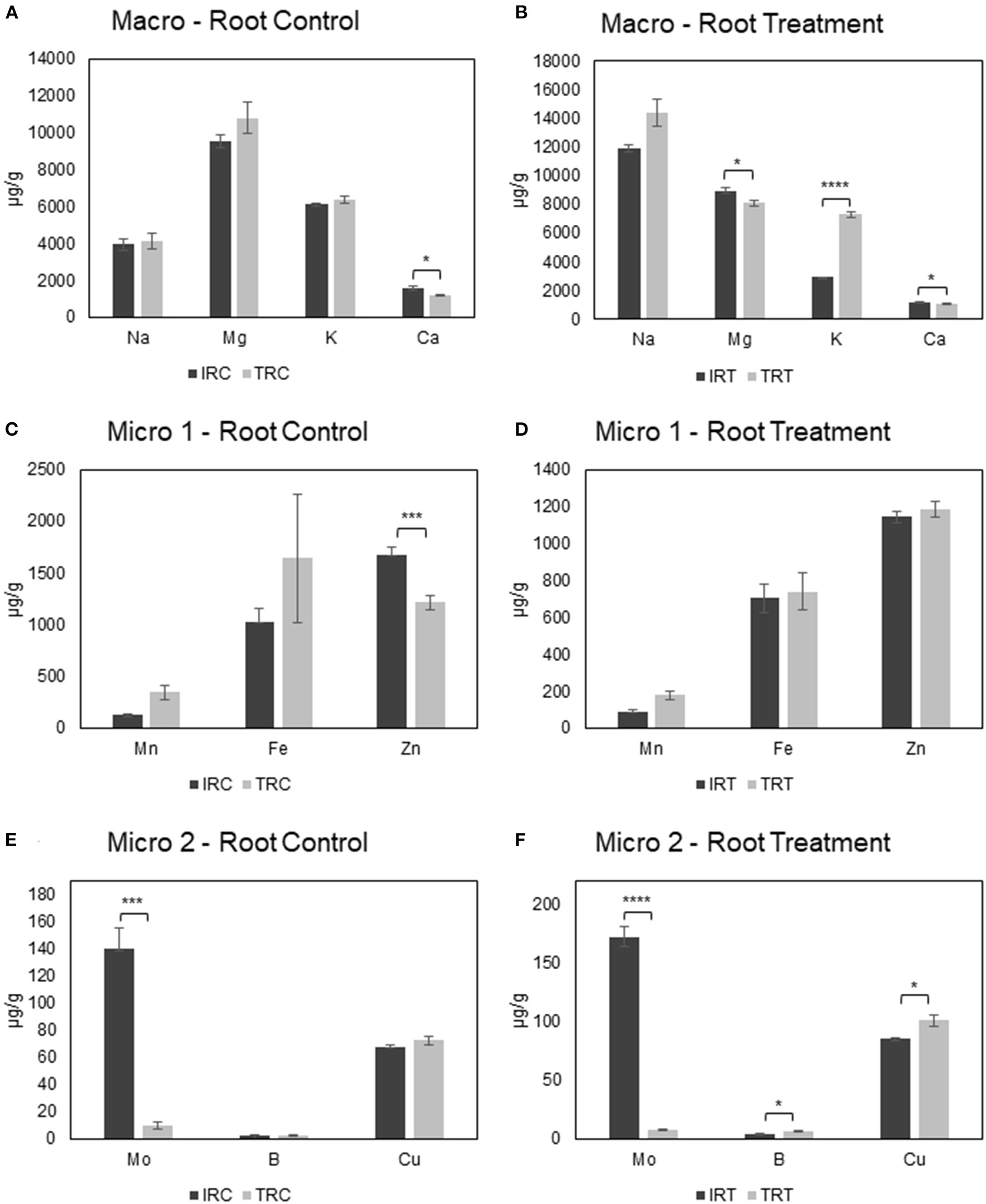

Supplement: Supplementary Figure 4 — Comparison of root ion contents in control and saline conditions. (A,B) Display the macroelement content comparisons for control and treatment conditions respectively. (C–F) Display the microelement content comparisosn for control and treatment conditions respectively. *p < 0.05; **p < 0.01; ***p < 0.005; ****p < 0.001. [file Image_4.tif]
